# Evaluation of the Properties of PHB Composite Filled with Kaolin Particles for 3D Printing Applications Using the Design of Experiment

**DOI:** 10.3390/ijms232214409

**Published:** 2022-11-19

**Authors:** Přemysl Menčík, Radek Přikryl, Štěpán Krobot, Veronika Melčová, Soňa Kontárová, Roderik Plavec, Jan Bočkaj, Vojtech Horváth, Pavol Alexy

**Affiliations:** 1 Institute of Material Chemistry, Faculty of Chemistry, Brno University of Technology, Purkyňova 464/118, 612 00 Brno, Czech Republic; 2 Institute of Natural and Synthetic Polymers, Faculty of Chemical and Food Technology, Slovak University of Technology in Bratislava, Radlinského 9, 812 37 Bratislava, Slovakia

**Keywords:** 3D printing, FDM, PHB, composite, Design of Experiment, kaolin

## Abstract

In the presented work, poly(3-hydroxybutyrate)-PHB-based composites for 3D printing as bio-sourced and biodegradable alternatives to synthetic plastics are characterized. The PHB matrix was modified by polylactide (PLA) and plasticized by tributyl citrate. Kaolin particles were used as a filler. The mathematical method “Design of Experiment” (DoE) was used to create a matrix of samples for further evaluation. Firstly, the optimal printing temperature of the first and upper layers was determined. Secondly, the 3D printed samples were tested with regards to the warping during the 3D printing. Testing specimens were prepared using the determined optimal printing conditions to measure the tensile properties, impact strength, and heat deflection temperature (HDT) of the samples. The results describe the effect of adding individual components (PHB, PLA, plasticizer, and filler) in the prepared composite sample on the resulting material properties. Two composite samples were prepared based on the theoretical results of DoE (one with the maximum printability and one with the maximum HDT) to compare them with the real data measured. The tests of these two composite samples showed 25% lower warping and 8.9% higher HDT than was expected by the theory.

## 1. Introduction

Poly(3-hydroxybutyrate) is one of the most examined members of the polyhydroxyalkanoate polymer family. It is a highly crystalline, naturally sourced biopolymer synthesized by bacteria and biodegradable in an environment at ambient conditions [1]. PHB is a thermoplastic material with some of its properties, such as melting temperature, glass transition temperature, crystallinity, and tensile strength being very similar to polypropylene. However, PHB is significantly more brittle than PP [2]. It is also not well thermally stable [3]. Due to its high crystallinity and processing temperature, PHB has a high tendency to shrink or warp during 3D printing. Generally, warping could be reduced by the improvement of the adhesion of printed material to the printing bed or by setting the printing parameters precisely [4]. In recent years PHB has also had attention for its biocompatibility. With various 3D printing techniques on the rise, PHB is very much in use in the tissue engineering field. Concerning the FDM 3D printing of neat PHB, this usually turns out to have very poor results due to the significant brittleness of PHB. Several studies have investigated PHB and its copolymers as potential scaffold materials in a diverse range of tissue engineering applications [5,6]. A very effective approach to reduce warping and improve printability is the blending of PHB with another biopolymer or the addition of filler. PHB is miscible with polyethylene oxide, polyvinyl acetate, polymethyl methacrylate, polycaprolactone, or polylactic acid at different ratios and temperatures [7].

Polylactide (PLA) is well known for its biocompatibility and compostability [8], and it is also well-compatible with PHB, enhancing the 3D printing properties of the mixture [9]. It is characterized by high tensile strength, tensile modulus, and low elongation, which makes it more than suitable for load-bearing applications [10]. Neat PHB, PHB-PLA, or other PLA blends are used in medicine for the 3D printing of scaffolds and hard tissue substitutes [11,12,13]. Up to now, PLA is also the most widely used material for 3D printing by FDM in terms of biodegradable and nonbiodegradable materials.

Palm fibres, lignin, or cellulose nanofibrils may be used as an organic filler, which decreases the warping of the PHB and, at the same time, does not influence biodegradability [14,15,16,17]. Many kinds of inorganic fillers, such as iron and copper particles, have been used to reduce thermal expansion and increase thermal conductivity for other technical plastic matrices [18,19]. Short glass or carbon fibres are suitable for enhancing mechanical properties [20,21]. Montmorillonite nanoclay has also been used to increase mechanical properties [22]. Many other materials like alumina, Zn, and Ti oxides can also be utilized [23]. Several previous works focusing on the evaluation of the influence of other inorganic fillers have also demonstrated kaolin as a usable filler for the bio-source PHB/PLA matrix composites used for 3D printing. The mechanical properties and printability of the kaolin-filled composite do not deteriorate, while differential scanning calorimetry proves there is no degradable effect of the kaolin on the matrix [24]. In another study, kaolin was used to enhance the flow properties of the plastic and showed the potential to be used as a rheological modifier [25].

In some studies, the influence of filler concentration on the mechanical properties of PHB composites has been determined [26], but not with respect to its printability. In others, the effect of printing parameters on tensile properties has been studied [27]. For an unambiguous choice of the appropriate composition of materials for 3D printing, knowledge of the effects of the individual components in a polymer mixture is essential. Design of Experiment (DoE) is one of the methods used to describe the influence of individual material components on the properties under study [28,29] or to predict the final mechanical properties [30]. In this work, the influence of kaolin filler, plasticizer, and PLA content on the mechanical, thermal, and 3D printing properties of PHB-based composites was established using the DoE method.

Ultimately, the Design of Experiment proved to be suitable for outlining the properties of composite material. Six optimal compositions (Comp_O_1 to Comp_O_6) were determined and it was confirmed that the calculated values are achievable after 3D printing and measuring of these compositions.

## 2. Results and Discussion

### 2.1. Optimization of 3D Printing Temperature

It was essential to find the optimal 3D printing temperature for all studied materials as they were used for 3D printing of the samples for all the following tests. The visually estimated temperatures from the temperature tower are summarized in Table 1. Optimal printing temperatures were mainly in the range of 175 to 185 °C.

The optimal printing temperatures for five reference samples (Comp_15 to Comp_20) were determined to be 180 ± 5 °C. The deviation of 5 °C was caused by the temperature regulation of the 3D printer.

Since the evaluation of the precision of geometric elements is only visual, the results are relatively highly burdened by the observer’s individuality. In the steps to follow, the value of optimal temperature will be more specified.

### 2.2. Determination of Warping Coefficient

Before the warping coefficient of all examined samples could be determined, it was necessary to find the printing temperature of the first layer. The printing temperature of the first layer is one of the most crucial parameters of FDM 3D printing. The first layer is the only one in direct contact with the printing bed and may have a different temperature (*T*_1_) from the rest of the printed layers (*T*_2_). The *T*_1_ temperature must be high enough to ensure that the first layer adheres perfectly to the printing bed. On the other hand, it must not be too high to prevent excessive melting and degradation of the printed material. The *T*_2_ temperature is usually lower for PLA-like materials to maintain a satisfactory shape of the printed part and avoid shrinkage. In addition, due to the low thermal stability of PHB, degradation is prevented. 

**Figure 1:**
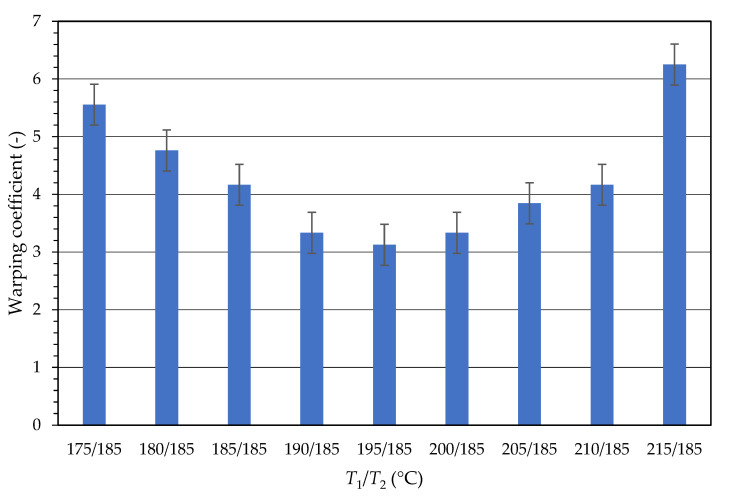
The dependence of the first-layer temperature (*T_1_*) on the warping coefficient of the material.

A warping test with variable temperature *T*_1_ and fixed temperature *T*_2_ = 185 °C was conducted on the sample Comp_21, where *T*_2_ was obtained from the temperature tower. Figure 1 shows the dependence of the warping coefficient on the printing temperature of the first layer (*T*_1_). The results showed that the optimal printing temperature *T*_1_ in this case equalled 195 °C when the warping coefficient reached the lowest values, and thus, the influence of warping was at a minimum. Below this temperature, the polymer melt had a low viscosity and did not adhere sufficiently to the printing bed. On the other hand, above the optimum temperature, there was already a large difference between *T*_1_ and *T*_2_. The printer´s extruder did not have time to cool down and, therefore, printed several layers on top of each other at a higher temperature than *T*_2_. This caused deformation of the bottom of the printed specimen and intensified warping. Temperature *T*_1_ was used further for all tested samples.

Test of warping during 3D printing was performed for all samples and all temperatures in the range of their optimal printing temperatures. The resulting temperatures with the lowest warping coefficient for each sample are presented in Table 2. From the warping test, the optimal printing temperature was further specified and the warping coefficient was calculated. Table 2 also shows that regarding the warping coefficient values, the optimal printing temperature for sample Comp_21 was 190 °C. This was a slightly higher temperature than the one used in the previous test, but this deviation was to determine whether the optimal printing temperature *T*_1_ was negligible.

Temperatures obtained from the warping test were used for 3D printing of the testing samples for all following tests. The optimal printing temperatures were relatively close together, which indicates that they were not significantly dependent on the ratio between the individual components in the composite samples.

### 2.3. Tensile Test

Young’s modulus of elasticity *E* and tensile strength *σ* values were obtained from the tensile test of the samples. The results of elongation at break were burdened with a significant standard deviation and therefore were not published.

**Figure 2:**
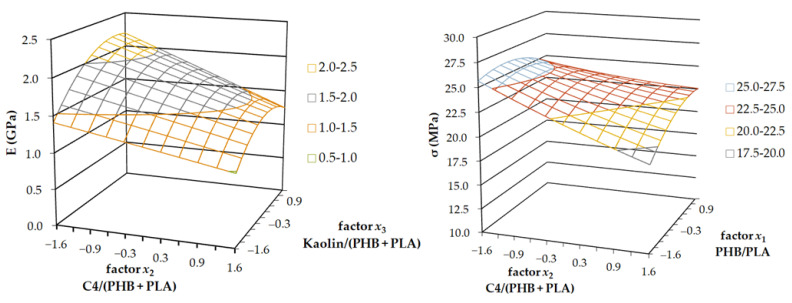
The 3-axis plots of the tensile test results; Young’s modulus of elasticity (**left**) and tensile strength (**right**).

The results of Young’s modulus report showed that all three DoE factors were statistically relevant. The lowest level of significance was for parameter *b*_1_. Therefore, factor *x*_1_ was fixed on coded level 0, which means the middle of its range. Figure 2a shows the plot of dependence of Young’s modulus of elasticity on the sample’s composition represented by factors *x*_2_ and *x*_3_. With increasing plasticiser content, e.g., with increasing factor *x*_2_, Young’s modulus linearly decreased. With increasing kaolin content in the material, e.g., with increasing factor *x*_3_, modulus *E* also increased until it reached a maximum. Further increase of kaolin content did not affect the modulus *E* increase, and the value of *E* was relatively constant beyond this maximum point. Filling with kaolin resulted in a toughening effect, and the *E* value increased up to this kaolin concentration. Also, the viscosity of the melted polymer during 3D printing was too high, and the connection of the printed layers was weaker beyond this point. Moreover, the high viscosity of polymer melt during 3D printing created larger voids, which decreased thermal stability (HDT). This trend was also confirmed in a full range of factor *x_1_,* e.g., in ranges of PLA content.

The results of the tensile strength report showed that only parameter *b_2_* was statistically relevant (content of plasticizer C4), whereas the other two parameters were not. The lowest level of significance was shown by parameter *b_3_,* and therefore factor *x_3_* was fixed on coded level 0. The plot of dependence of tensile strength *σ* on the DoE factors *x*_2_ and *x*_1_ is presented in Figure 2b. Although, since factor *x*_1_ was not statistically relevant, we cannot evaluate and comment on its influence on tensile strength. The tensile strength decreased linearly with the increasing content of C4 in the mixture. This result indicated the functioning plasticization ability of C4, which caused plastic deformation to occur at even lower loads. The shape of the stress-strain curve showed normative shape A, which means that the first local maximum corresponded with the tensile strength of the sample.

### 2.4. Unnotched and Notched Charpy Impact Test

**Figure 3:**
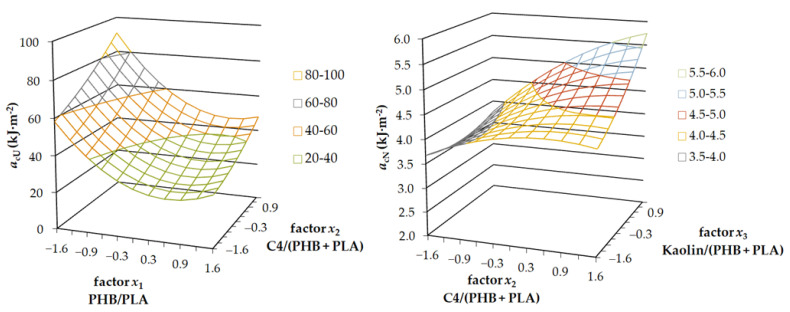
The 3-axis plots of the unnotched (**left**) and the notched (**right**) Charpy impact test results.

The unnotched Charpy impact test results implied that the factors *x*_1_ and *x*_2_ were statistically relevant. Interestingly, although factor *x*_3_ corresponded with kaolin content, it was not statistically relevant and thus fixed on coded level 0. The graph in Figure 3a shows the dependence of unnotched impact strength (*a*_cU_) on the content of plasticiser, e.g., factor *x*_2_, which increased linearly. This result again confirmed the proper functioning of plasticizer C4 in the samples. The highest value of impact strength was measured for the lowest content of PHB in the mixture. The dependence of impact strength on PHB content was non-linear. The lowest measured value was located on coordinate *x*_1_ = 0.6, and from this point on, it increased slightly again. The further increase of *a*_cU_ values could have been caused by the weaker connections of the 3D printed layers at higher filler contents.

The plot for the notched Charpy impact test, which is illustrated in Figure 3b, is somewhat different than for the unnotched. In this case, the statistically relevant factors were *x*_2_ and *x*_3_. Factor *x*_1_ was not relevant and fixed on coded level 0. The value of notched impact strength (*a*_cN_) increased non-linearly with the increasing content of the plasticiser. It also increased with the increasing content of kaolin particles. This effect was, however, more relevant for the higher content of plasticiser because of better dispersion of the particles in the plasticized matrix, which corresponded with slower crack propagation.

### 2.5. Heat Deflection Temperature Test

**Figure 4:**
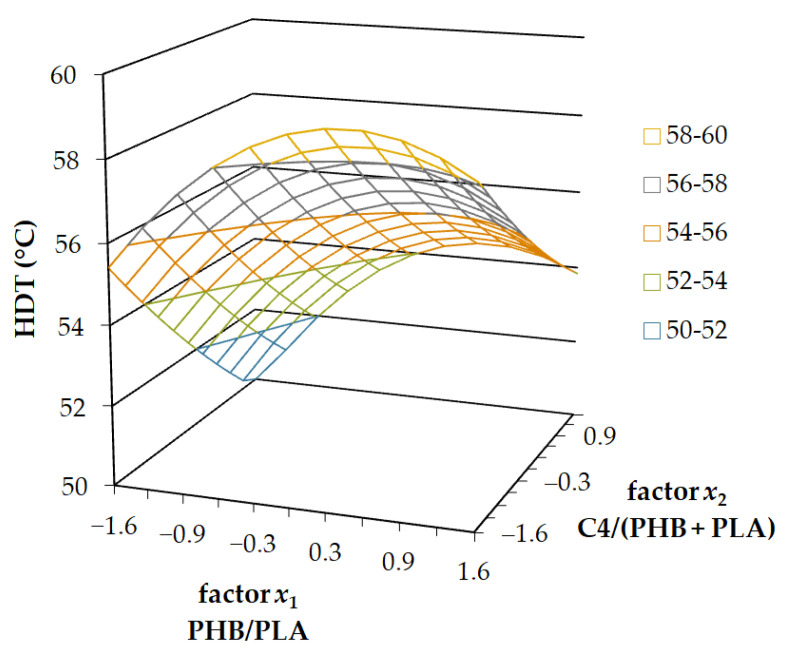
The 3-axis plot of the Heat Deflection Temperature Test results at 1.82 MPa load.

The Heat Deflection Temperature (HDT) Test reported all the factors as statistically irrelevant, with *x*_3_ being the least significant of all. Increasing the filling concentration with kaolin showed a smooth linear improving effect on the HDT, but the overall effect was negligible. Factor *x*_3_ was fixed on coded level 0, and the plot was created using factors *x*_1_ and *x*_2_. The decreasing trend of the HDT with increasing content of plasticizer C4 (factor *x*_2_) is evident from the results presented in Figure 4. The dependence of the HDT on factor *x*_1_ was non-linear, with the maximum point in coded level 0.4. The HDT decreased beyond this point, probably because of weaker connections of the 3D printed layers. This non-linear trend was less significant at higher kaolin contents.

### 2.6. Optimal Composition of Samples

Regarding the DoE results, the optimal composition of the composite samples, which should, by theory, achieve the maximum value of measured property in each of the tests performed, was determined using the MS Excel Solver function. The optimal material compositions of the samples (Comp_O_1 to Comp_O_6) and the theoretical maximum values of the measured properties are presented in coded levels in Table 3. The material property aimed to maximize and is marked in bold for each composition.

Two samples were prepared to confirm that the optimal compositions calculated by theory to achieve the maximum values of measured properties corresponded with the real ones. Sample Comp_O_5 had optimal composition to achieve the lowest warping coefficient, and Comp_O_6 had the highest HDT. The material composition of Comp_O_5 and Comp_O_6 is given in Table 4.

Samples Comp_O_5 and Comp_O_6 were subjected to the Temperature Tower Test to obtain the range of usable printing temperatures. According to the results, the temperature 195 °C was chosen as the ideal printing temperature for both samples. All further testing specimens were printed at 195 °C for the first layer (*T*_1_) and also for all other layers (*T*_2_).

The analysis of the warping coefficient was performed on the mixture Comp_O_5 optimized for low warping. Five testing specimens were used for the test, and the mean value was compared with the theoretical value obtained from the DoE. The mixture Comp_O_6 was optimized for high-temperature stability and subjected to the HDT Test. Eight testing specimens were used for the test, and the mean value was compared with the theoretical value obtained from the DoE. Another six HDT testing specimens were annealed in a laboratory oven for 2 h at 80 °C to achieve another increasement in thermal stability. These samples were named An_Comp_O_6. All the results obtained are given in Table 5.

The obtained value of the warping coefficient was up to 25% lower than anticipated from the results of the DoE. This makes a difference of another two successfully printed layers within the printed sample. In terms of the HDT, the experimentally acquired values were up to 8.9% higher than the theoretical ones obtained from the DoE. It was even possible to increase the value of the HDT by up to 33.5% by annealing the sample. This occurred due to the secondary crystallization of PHB in the samples.

## 3. Materials and Methods

### 3.1. Materials

Poly(3-hydroxyburyrate) Biomer^®^ T22 (PHB), polylactid NatureWorks Ingeo^TM^ 4060D (PLA), and plasticizer Vertellus Citroflex^®^ 4 (C4) were used to prepare the polymer matrix. Kaolin Sedlec Ia was used as a filler. Both polymers were dried at 60 °C for 2 h before processing.

### 3.2. Sample Preparation

Composite blends were prepared by twin screw extruder, LabTech, Sorisole, Italy, with an L/D ratio of 40 and a screw diameter of 16 mm. The temperature profile of extrusion was 80/170/180/180/180/180/180/170/160/150 °C from feeder to die, and the speed of the screw was set to 100 RPM. The filament from the extruder was cooled in a water bath and cut by a strand pelletizer. All material granulates were dried before the next processing.

Filaments for 3D printing were prepared by a single screw extruder, Thermo Fischer Scientific, Waltham, MA, USA, with an L/D ratio of 25. The temperature profile of extrusion was 185/180/175/170 °C from feeder to die, and the speed of the screw was set to 25 RPM. The extruded filament was cooled down in the water bath and winded on spools by a custom-made filament winder.

All testing samples were 3D printed by FDM printer Prusa i3MK3 controlled by V3.9.0 firmware version (Prusa Research a.s., Prague, Czech Republic), and using 0.4 mm E3D V6 nozzle.. All G-codes were prepared in PrusaSlicer, software version 1.41.2. PrusaSlicer is based on an open-source platform Slic3r, which is licensed under the GNU Affero General Public License, version 3. The layer height was 0.2 mm. The printing speed was set to 40 mm/s for perimeters, and 60 mm/s for rectilinear infill. The printing speed of the first layer was 70% of the printing speed of the other layers. The infill density was set to 100% to create the entirely filled samples. The print profile used was taken from the profile for PLA. Therefore, the printed sample was cooled by a fan at 100% from the second layer.

### 3.3. Optimization of 3D Printing Temperature

The Temperature Tower Test (TTT) samples were printed from each prepared composite blend [31]. They consisted of 6 floors, where each floor was made up of 8 different geometric elements. The 3D printing began at 220 or 195 °C, and each consecutive floor above the previous one was printed at a temperature 5 °C lower. The geometric elements from each floor were evaluated visually to determine the optimal printing temperature.

### 3.4. Determination of Warping Coefficient

Warping is a negative effect manifested by a warping coefficient. The warp testing samples [31] were printed for warping analysis. The height of the printed sample was observed during printing to the point when the specimen began to warp and detach from the printing bed. The theoretical total height of the sample (10 mm) was divided by the maximum height reached by the sample. The resulting value is the warping coefficient. This relation is shown in Equation (1).

(1) $$Warping\;coefficient=\frac{Total\;theoretical\;height\;of\;the\;sample\;\left(10\;mm\right)}{Maximum\;height\;reached\;by\;the\;sample}$$

### 3.5. Tensile Test

Standardised double-paddle testing specimens (dogbones 5A with cross Section 4 × 2 mm) were 3D printed according to the ČSN EN ISO 527-2. The tensile test was performed using the universal testing machine ZWICK Z010 with a 10 kN load cell. The test speed of the measurement was set to 5 mm/min in the deformation range of 0.05–0.25% to determine Young’s modulus and to 50 mm/min for the rest of the tests performed. The dogbone 5A test samples were 3D printed with a longitudinally oriented printing trajectory [31]. The number of tested specimens was at least 5 for each tested series.

### 3.6. Unnotched and Notched Charpy Impact Test

Both tests of impact strength were performed using CEAST Resil Impactor Junior with pendulum hammer 2.7 J. The rectangular testing samples were 3D printed according to ISO 179. The 2 mm deep V-notch with a tip radius of 0.25 mm radius machined on one face was made using a CEAST Power-Driven Notchvis cutting machine. Samples were tested in the edgewise position.

### 3.7. Heat Deflection Temperature Test

The Heat Deflection Temperature (HDT) Test was performed according to the ČSN EN ISO 294-4 standard using a custom-made laboratory testing machine. Samples for the test were 3D printed and measured in a flatwise position. The samples after 3D printing could not be measured at the lower load allowed by the standard (0.455 MPa) because of the internal tension and therefore were measured using a 1.82 MPa load only.

### 3.8. Design of Experiment—Composition of Samples

To cover the broadest possible range of resulting material properties the 3-factor, 5-level Design of Experiment (DoE) was compiled. The following limits were chosen:

- The content of PHB ranged from 60 to 95 wt%
- The content of plasticizer C4 ranged from 8 to 15 wt%
- The content of kaolin filler ranged from 5 to 20 wt%

The first factor of the experiment (*x*_1_) was the PHB to PLA ratio, the second factor (*x*_2_) was the plasticizer to total polymer content ratio (C4/(PHB + PLA)), and the third factor (*x*_3_) was the filler to total polymer content ratio (Kaolin/(PHB + PLA)). The limit values, mean values, and steps of the experiment in coded and real values are listed in Table 6. The limit values in coded levels −1.682 and 1.682 correspond with the limit composition of the samples. The mean value is located on the coded coordinate 0. The confidence interval was 95%.

Subsequently, the mathematical matrix of samples was built from the limit values in (from limits in Table 6). The values of factors in coded and real levels are summarized in Table 7. The samples starting with Comp_15 up to Comp_20 have the same material composition and provide information about the repeatability and accuracy of the tests. They were tested in random order with all other samples, and the statistical relevance of measurement was established from the results.

Regarding the given limit values and DoE factors, it was possible to determine the final composition of all testing samples. The actual sample composition is listed in Table 8. The sample Comp_21, not containing the filler, was added to the list for the first estimation of 3D printing conditions.

Experimental data were plotted in 3D wave graphs. The graphs show the dependence of the measured value on two statistically relevant DoE factors. The third, less relevant or even irrelevant factor was fixed at a constant level coded as 0. Equation (2) was used to plot the graphs. Parameters *x*_1_, *x*_2_, and *x*_3_, represent DoE factors, PHB/PLA, C4/(PHB + PLA), and Kaolin/(PHB + PLA) respectively.

*y* = *b*_0_ + *b*_1_*x*_1_ + *b*_2_*x*_2_ + *b*_3_*x*_3_ + *b*_11_*x*_1_^2^ + *b*_22_*x*_2_^2^ + *b*_33_*x*_3_^2^ + *b*_12_*x*_1_*x*_2_ + *b*_13_*x*_1_*x*_3_ + *b*_23_*x*_2_*x*_3_ (2)

Statistically relevant parameters *b_i_* and thus relevant factors *x_i_* were calculated for each test from the mathematical analysis of standard deviations of the measurements and critical values.

## 4. Conclusions

Poly(3-hydroxybutyrate) (PHB)-based composites for 3D printing were successfully prepared in the presented work. A mathematical matrix of samples with different material compositions was designed based on the Design of Experiment (DoE) method. The basic range of the usable printing temperatures was determined by the Temperature Tower Test. The optimal printing temperature of the first layer (*T*_1_) was found to be 195 °C. It was determined by a warping test performed during 3D printing and chosen as the optimal printing temperature for all samples.

The testing specimens were 3D printed under the found optimal conditions. The mechanical properties of the composites improved with increasing filler content (kaolin particles) until they reached the maximum point. The polymer melt showed too high a viscosity to achieve the optimal connection of the printed layers beyond this point during 3D printing. With further increasements of the filler content, the mechanical properties remained either constant or even deteriorated. Tensile strength and the heat deflection temperature (HDT) deteriorated with increasing kaolin content, but on the other hand, the printability of materials, represented by a low warping coefficient, improved.

In the end, six optimal compositions (Comp_O_1 to Comp_O_6) were established to provide the highest Young´s modulus, tensile strength, unnotched, and notched impact strength, warping coefficient, and HDT, based on the results of the DoE.

Two material compositions calculated by theory with minimum warping coefficient and maximum HDT (Comp_O_5 and Comp_O_6) were prepared based on the DoE results and 3D printed to confirm that the theoretically determined highest values of the examined properties of these compositions were achievable after their measurement. These samples performed up to 25% lower warping coefficient and up to 8.9% higher HDT after they were measured than was anticipated from the theoretical results calculated by the DoE. Another increasement of HDT, by up to 33.5%, was achieved by annealing the samples (An_Comp_O_6).

In conclusion, the Design of Experiment proved to be suitable for outlining the properties of composite material as it consisted of many components, which could vary in their content. Six optimal compositions were determined and it was confirmed that the calculated values were achievable after 3D printing the compositions and measuring them. The DoE method saves much time and raw materials, as several samples with the most optimal properties can be prepared, and there is no need to prepare and test all of the possibilities.

## Figures and Tables

**Table 1:** The range of optimal printing temperatures of composites observed from the temperature tower.

Sample	Optimal Printing Temperature (°C)	Sample	Optimal Printing Temperature (°C)
Comp_1	185–175	Comp_11	180–175
Comp_2	180–175	Comp_12	180–175
Comp_3	175 and 200	Comp_13	200
Comp_4	200–195	Comp_14	180–175
Comp_5	200–195 and 180–175	Comp_15	180–175
Comp_6	200 and 180–175	Comp_16	180–175
Comp_7	200–195 and 175	Comp_17	185–175
Comp_8	200–195 and 180–175	Comp_18	180–175
Comp_9	200–175	Comp_19	175
Comp_10	175	Comp_20	175
		Comp_21	200–175

**Table 2:** The optimal composite printing temperatures based on the warping test and warping coefficient.

Sample	Printing Temperature *T*_2_ (°C)	Warping Coefficient	Sample	Printing Temperature *T*_2_ (°C)	Warping Coefficient
Comp_1	185	3.2 ± 0.1	Comp_11	185	4.5 ± 0.0
Comp_2	185	4.0 ± 0.2	Comp_12	185	5.0 ± 0.0
Comp_3	195	3.5 ± 0.2	Comp_13	190	3.7 ± 0.2
Comp_4	185	7.1 ± 0.0	Comp_14	185	4.8 ± 0.3
Comp_5	185	4.0 ± 0.2	Comp_15	185	4.2 ± 0.0
Comp_6	185	6.7 ± 0.6	Comp_16	195	4.6 ± 0.6
Comp_7	185	4.4 ± 0.3	Comp_17	185	4.0 ± 0.2
Comp_8	185	6.7 ± 0.6	Comp_18	185	4.4 ± 0.3
Comp_9	185	1.3 ± 0.0	Comp_19	185	4.0 ± 0.2
Comp_10	185	4.0 ± 0.2	Comp_20	185	4.2 ± 0.0
			Comp_21	190	1.5 ± 0.0

**Table 3:** The optimal compositions of samples according to the DoE results.

Factor/Property	Comp_O_1	Comp_O_2	Comp_O_3	Comp_O_4	Comp_O_5	Comp_O_6
*x*_1_	0.369	−0.049	−1.682	−1.682	−1.682	0.436
*x*_2_	−1.682	−1.682	1.682	1.682	−0.141	−1.682
*x*_3_	1.200	1.682	−1.682	1.682	−0.739	1.682
*E* (GPa)	**2.3**	2.2	0.4	0.8	1.2	2.2
*σ* (MPa)	26.2	**26.4**	18.6	19.5	22.4	26.3
*a*_cU_ (kJ/m^2^)	21.8	22.9	**109.0**	83.3	79.2	21.7
*a*_cN_ (kJ/m^2^)	3.6	3.7	4.2	**5.9**	4.3	3.7
Warping coefficient	14.3	25.0	2.1	2.2	**1.6**	50.0
HDT (°C)	60.9	61.3	48.4	50.1	51.4	**61.5**

**Table 4:** The actual weight percentage composition of the prepared samples.

wt%	Comp_O_5	Comp_O_6
PHB	48.6	67.3
PLA	32.3	7.7
C4	10.4	9.3
Kaolin	8.7	15.7

**Table 5:** The results of the warping test and the HDT Test of optimised mixtures.

Sample	Warping Coefficient	Warping Coefficient by DoE	HDT (°C)	HDT by DoE (°C)
Comp_O_5	1.3 ± 0.1	1.6	-	-
Comp_O_6	-	-	64.3 ± 2.7	61.5
An_Comp_O_6	-	-	76.0 ± 6.1	-

**Table 6:** Limit values of DoE and steps in coded and real levels.

	Limit Values of the Experiment					
Factors	−1.682	−1	0	1	1.682	Step
*x*_1_-PHB/PLA	1.5000	5.0476	10.2490	15.4510	18.9990	5.202
*x*_2_-C4/(PHB/PLA)	0.0870	0.1051	0.1317	0.1583	0.1765	0.027
*x*_3_-Kaolin/(PHB/PLA)	0.0526	0.0925	0.1509	0.2094	0.2492	0.058

**Table 7:** The composition of the samples in coded and real levels of DoE.

	Factors in Coded Levels			Factors in Real Levels		
Sample	PHB/PLA	$\frac{C4}{\left(PHB+PLA\right)}$	$\frac{Kaolin}{\left(PHB+PLA\right)}$	PHB/PLA	$\frac{C4}{\left(PHB+PLA\right)}$	$\frac{Kaolin}{\left(PHB+PLA\right)}$
Comp_1	−1	−1	−1	5.0476	0.1051	0.0925
Comp_2	1	−1	−1	15.4510	0.1051	0.0925
Comp_3	−1	1	−1	5.0476	0.1583	0.0925
Comp_4	1	1	−1	15.4510	0.1583	0.0925
Comp_5	−1	−1	1	5.0476	0.1051	0.2094
Comp_6	1	−1	1	15.4510	0.1051	0.2094
Comp_7	−1	1	1	5.0476	0.1583	0.2094
Comp_8	1	1	1	15.4510	0.1583	0.2094
Comp_9	−1.682	0	0	1.5000	0.1317	0.1509
Comp_10	1.682	0	0	18.9990	0.1317	0.1509
Comp_11	0	−1.681	0	10.2490	0.0870	0.1509
Comp_12	0	1.681	0	10.2490	0.1765	0.1509
Comp_13	0	0	−1.681	10.2490	0.1317	0.0526
Comp_14	0	0	1.681	10.2490	0.1317	0.2492
Comp_15-20	0	0	0	10.2490	0.1317	0.1509

**Table 8:** The actual composition of testing samples.

Sample	PHB (wt%)	PLA (wt%)	C4 (wt%)	Kaolin (wt%)
Comp_1	69.7	13.8	8.8	7.7
Comp_2	78.4	5.1	8.8	7.7
Comp_3	66.7	13.2	12.7	7.4
Comp_4	75.1	4.9	12.7	7.4
Comp_5	63.5	12.6	8.0	15.9
Comp_6	71.5	4.6	8.0	15.9
Comp_7	61.0	12.1	11.6	15.3
Comp_8	68.7	4.4	11.6	15.3
Comp_9	46.8	31.2	10.3	11.8
Comp_10	74.1	3.9	10.3	11.8
Comp_11	73.6	7.2	7.0	12.2
Comp_12	68.6	6.7	13.3	11.4
Comp_13	76.9	7.5	11.1	4.4
Comp_14	66.0	6.4	9.5	18.0
Comp_15-20	71.0	6.9	10.3	11.8
Comp_21	60.9	26.1	13.0	0

## Data Availability

Not applicable.
